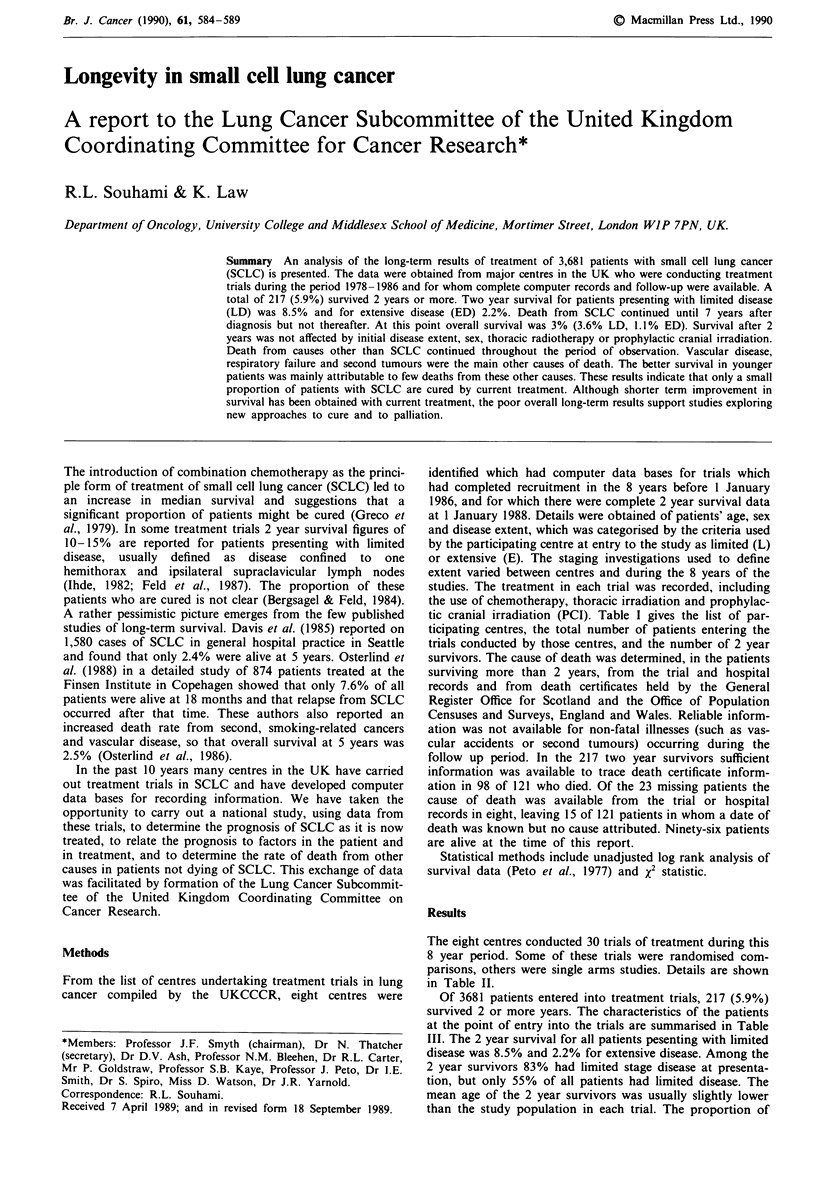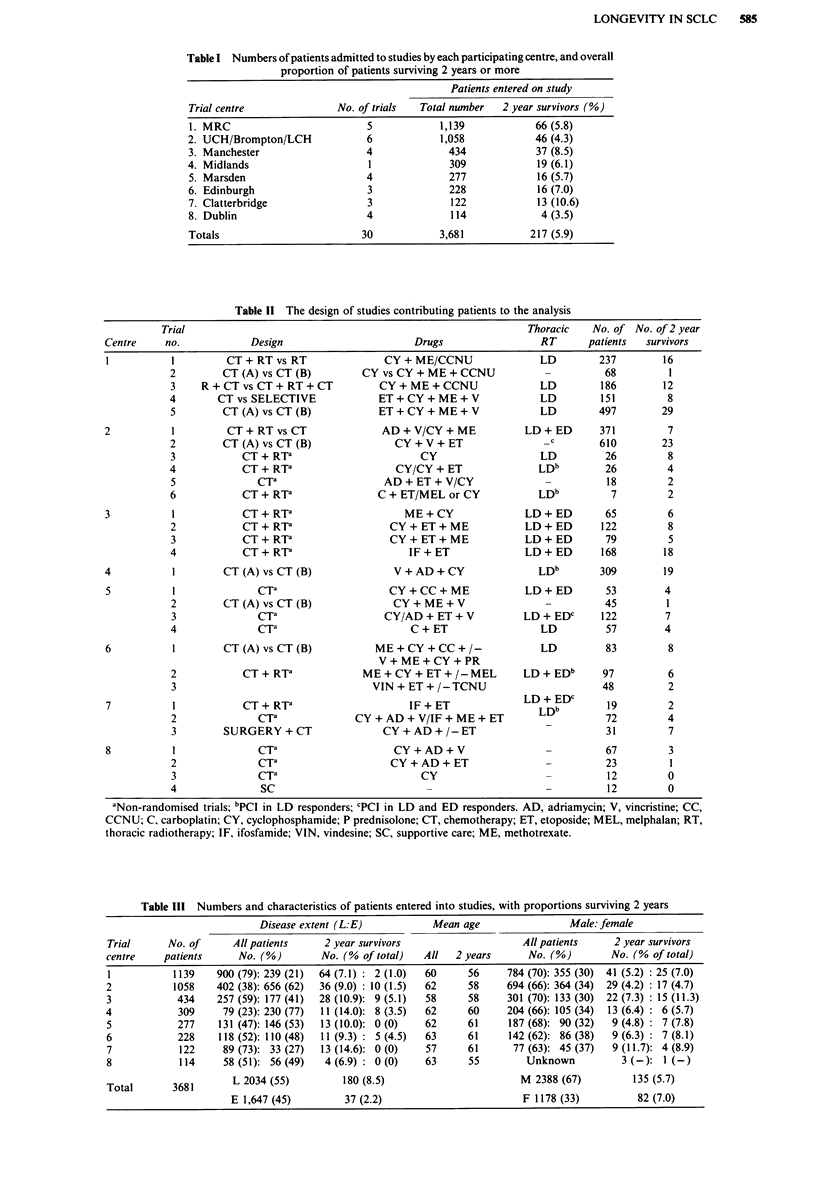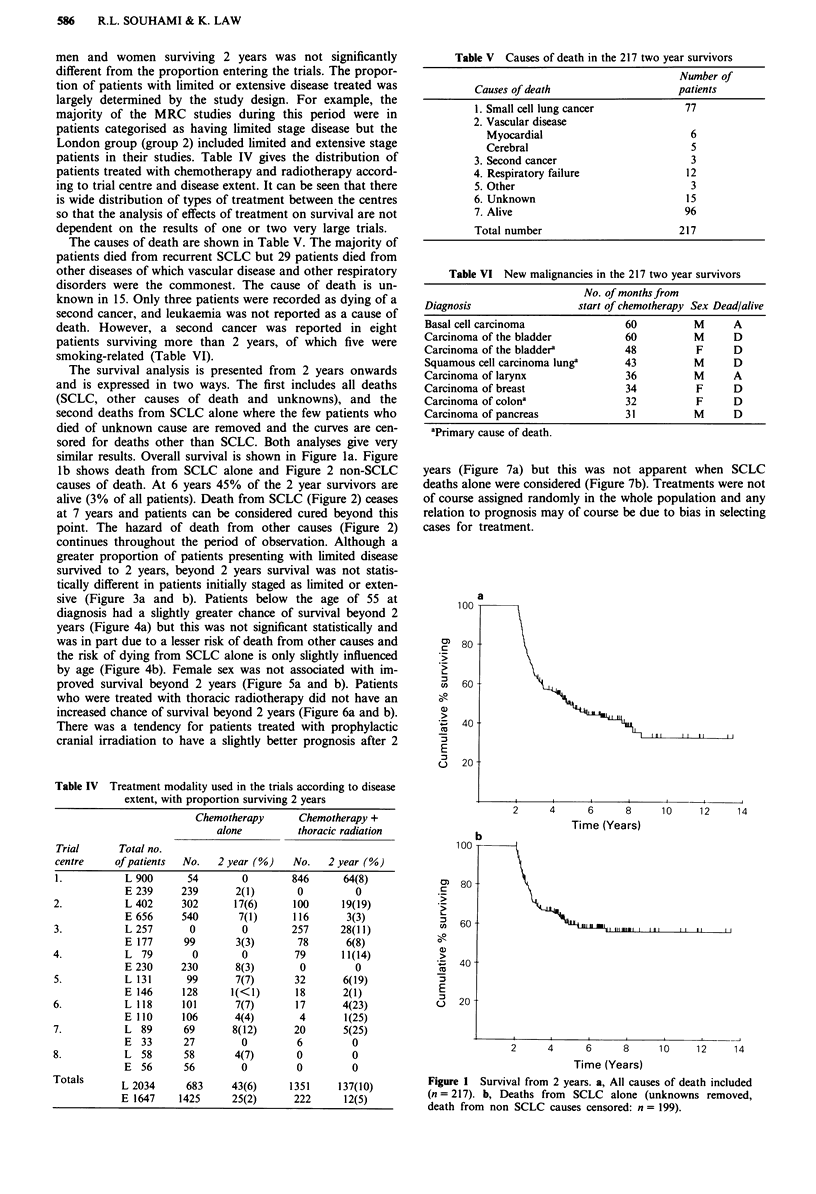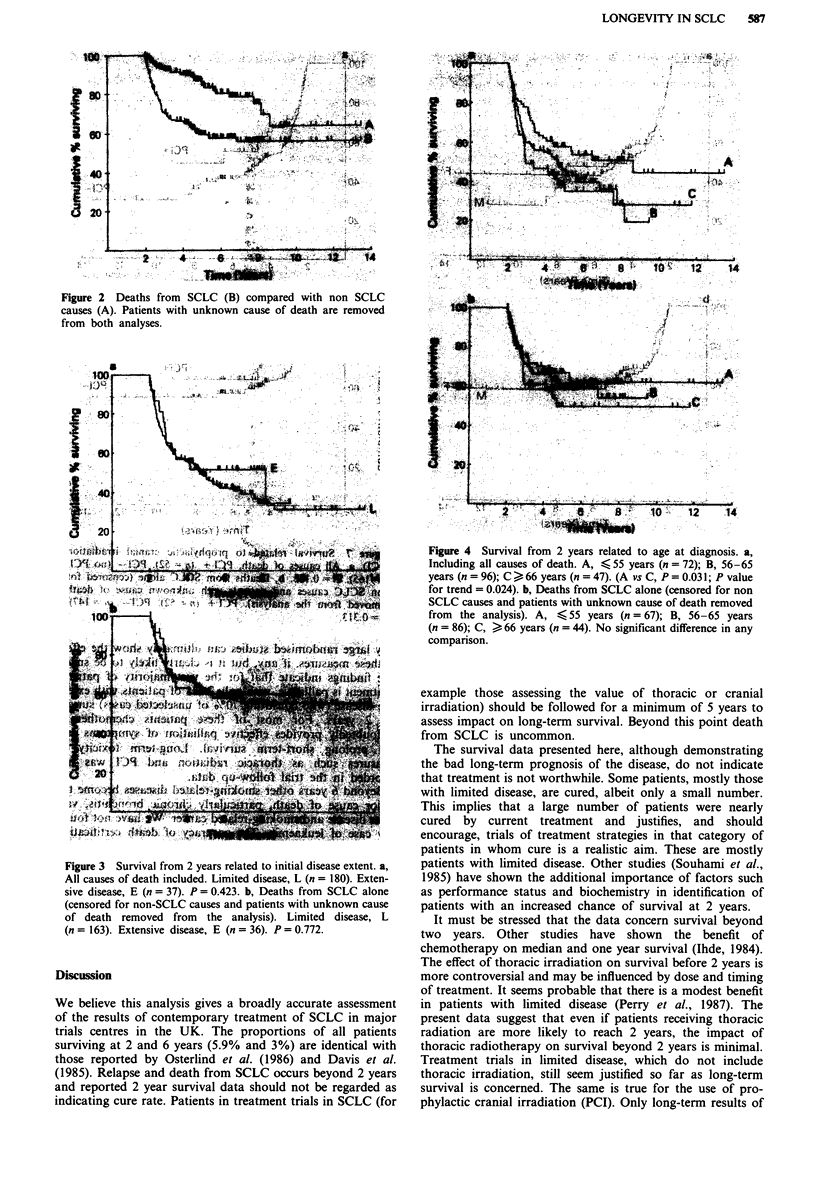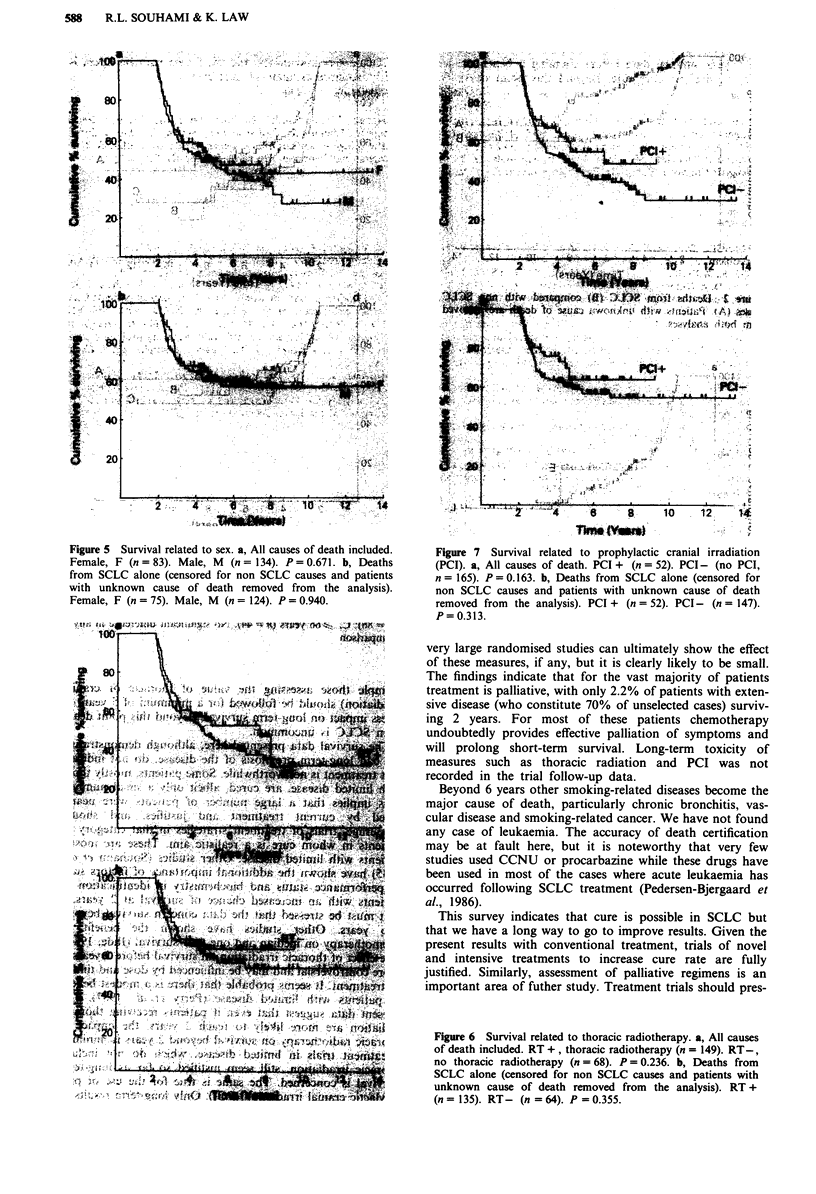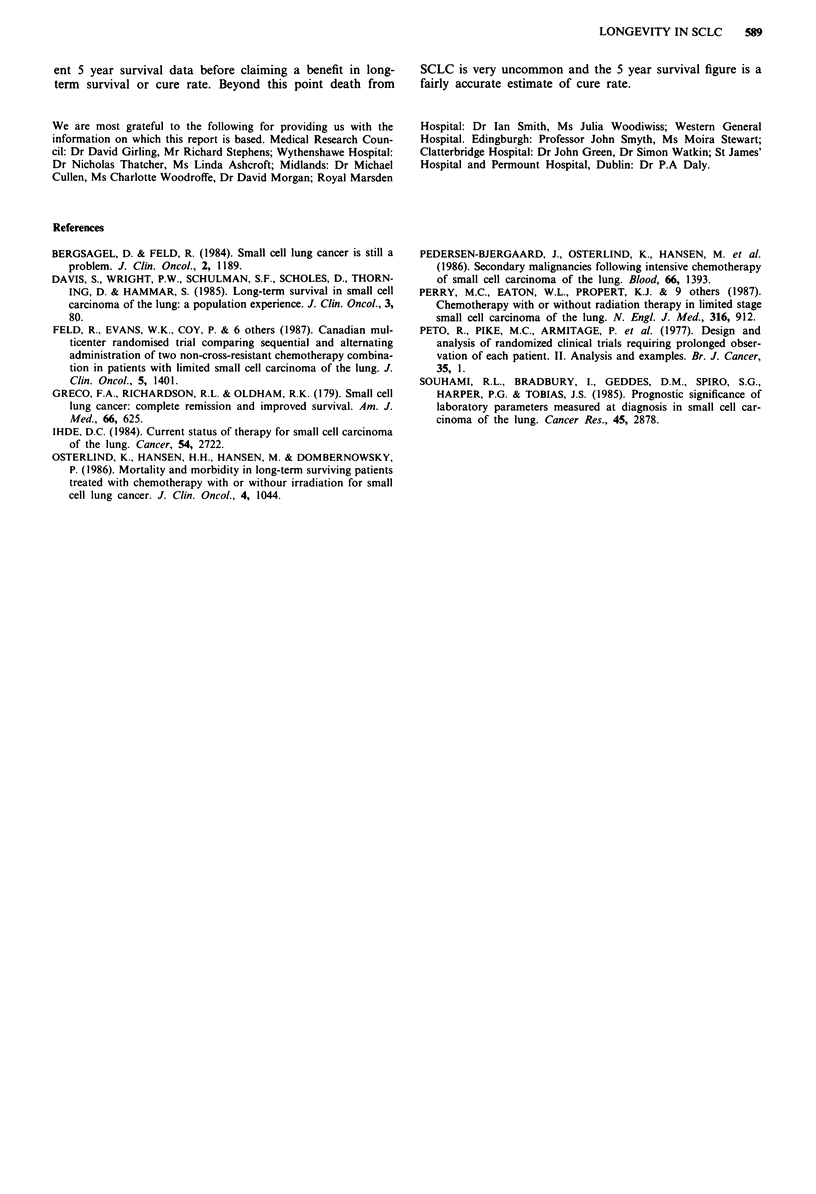# Longevity in small cell lung cancer. A report to the Lung Cancer Subcommittee of the United Kingdom Coordinating Committee for Cancer Research.

**DOI:** 10.1038/bjc.1990.131

**Published:** 1990-04

**Authors:** R. L. Souhami, K. Law

**Affiliations:** Department of Oncology, University College and Middlesex School of Medicine, London, UK.

## Abstract

An analysis of the long-term results of treatment of 3,681 patients with small cell lung cancer (SCLC) is presented. The data were obtained from major centres in the UK who were conducting treatment trials during the period 1978-1986 and for whom complete computer records and follow-up were available. A total of 217 (5.9%) survived 2 years or more. Two year survival for patients presenting with limited disease (LD) was 8.5% and for extensive disease (ED) 2.2%. Death from SCLC continued until 7 years after diagnosis but not thereafter. At this point overall survival was 3% (3.6% LD, 1.1% ED). Survival after 2 years was not affected by initial disease extent, sex, thoracic radiotherapy or prophylactic cranial irradiation. Death from causes other than SCLC continued throughout the period of observation. Vascular disease, respiratory failure and second tumours were the main other causes of death. The better survival in younger patients was mainly attributable to few deaths from these other causes. These results indicate that only a small proportion of patients with SCLC are cured by current treatment. Although shorter term improvement in survival has been obtained with current treatment, the poor overall long-term results support studies exploring new approaches to cure and to palliation.


Br. J. Cancer (1990), 61, 584-589 © Macmillan Press Ltd., 1990

### Longevity in small cell lung cancer

### A report to the Lung Cancer Subcommittee of the United Kingdom Coordinating Committee for Cancer Research*

R.L. Souhami & K. Law

*Department of Oncology, University College and Middlesex School of Medicine, Mortimer Street, London W1P 7PN, UK.*

**Summary** An analysis of the long-term results of treatment of 3,681 patients with small cell lung cancer (SCLC) is presented. The data were obtained from major centres in the UK who were conducting treatment trials during the period 1978–1986 and for whom complete computer records and follow-up were available. A total of 217 (5.9%) survived 2 years or more. Two year survival for patients presenting with limited disease (LD) was 8.5% and for extensive disease (ED) 2.2%. Death from SCLC continued until 7 years after diagnosis but not thereafter. At this point overall survival was 3% (3.6% LD, 1.1% ED). Survival after 2 years was not affected by initial disease extent, sex, thoracic radiotherapy or prophylactic cranial irradiation. Death from causes other than SCLC continued throughout the period of observation. Vascular disease, respiratory failure and second tumours were the main other causes of death. The better survival in younger patients was mainly attributable to few deaths from these other causes. These results indicate that only a small proportion of patients with SCLC are cured by current treatment. Although shorter term improvement in survival has been obtained with current treatment, the poor overall long-term results support studies exploring new approaches to cure and to palliation.

The introduction of combination chemotherapy as the principle form of treatment of small cell lung cancer (SCLC) led to an increase in median survival and suggestions that a significant proportion of patients might be cured (Greco *et al.*, 1979). In some treatment trials 2 year survival figures of 10–15% are reported for patients presenting with limited disease, usually defined as disease confined to one hemithorax and ipsilateral supraclavicular lymph nodes (Ihde, 1982; Feld *et al.*, 1987). The proportion of these patients who are cured is not clear (Bergsagel & Feld, 1984). A rather pessimistic picture emerges from the few published studies of long-term survival. Davis *et al.* (1985) reported on 1,580 cases of SCLC in general hospital practice in Seattle and found that only 2.4% were alive at 5 years. Osterlind *et al.* (1988) in a detailed study of 874 patients treated at the Finsen Institute in Copehagen showed that only 7.6% of all patients were alive at 18 months and that relapse from SCLC occurred after that time. These authors also reported an increased death rate from second, smoking-related cancers and vascular disease, so that overall survival at 5 years was 2.5% (Osterlind *et al.*, 1986).

In the past 10 years many centres in the UK have carried out treatment trials in SCLC and have developed computer data bases for recording information. We have taken the opportunity to carry out a national study, using data from these trials, to determine the prognosis of SCLC as it is now treated, to relate the prognosis to factors in the patient and in treatment, and to determine the rate of death from other causes in patients not dying of SCLC. This exchange of data was facilitated by formation of the Lung Cancer Subcommittee of the United Kingdom Coordinating Committee on Cancer Research.

### Methods

From the list of centres undertaking treatment trials in lung cancer compiled by the UKCCCR, eight centres were identified which had computer data bases for trials which had completed recruitment in the 8 years before 1 January 1986, and for which there were complete 2 year survival data at 1 January 1988. Details were obtained of patients' age, sex and disease extent, which was categorised by the criteria used by the participating centre at entry to the study as limited (L) or extensive (E). The staging investigations used to define extent varied between centres and during the 8 years of the studies. The treatment in each trial was recorded, including the use of chemotherapy, thoracic irradiation and prophylactic cranial irradiation (PCI). Table I gives the list of participating centres, the total number of patients entering the trials conducted by those centres, and the number of 2 year survivors. The cause of death was determined, in the patients surviving more than 2 years, from the trial and hospital records and from death certificates held by the General Register Office for Scotland and the Office of Population Censuses and Surveys, England and Wales. Reliable information was not available for non-fatal illnesses (such as vascular accidents or second tumours) occurring during the follow up period. In the 217 two year survivors sufficient information was available to trace death certificate information in 98 of 121 who died. Of the 23 missing patients the cause of death was available from the trial or hospital records in eight, leaving 15 of 121 patients in whom a date of death was known but no cause attributed. Ninety-six patients are alive at the time of this report.

Statistical methods include unadjusted log rank analysis of survival data (Peto *et al.*, 1977) and $\chi^2$ statistic.

### Results

The eight centres conducted 30 trials of treatment during this 8 year period. Some of these trials were randomised comparisons, others were single arms studies. Details are shown in Table II.

Of 3681 patients entered into treatment trials, 217 (5.9%) survived 2 or more years. The characteristics of the patients at the point of entry into the trials are summarised in Table III. The 2 year survival for all patients pesenting with limited disease was 8.5% and 2.2% for extensive disease. Among the 2 year survivors 83% had limited stage disease at presentation, but only 55% of all patients had limited disease. The mean age of the 2 year survivors was usually slightly lower than the study population in each trial. The proportion of

*Members: Professor J.F. Smyth (chairman), Dr N. Thatcher (secretary), Dr D.V. Ash, Professor N.M. Bleehen, Dr R.L. Carter, Mr P. Goldstraw, Professor S.B. Kaye, Professor J. Peto, Dr I.E. Smith, Dr S. Spiro, Miss D. Watson, Dr J.R. Yarnold.
Correspondence: R.L. Souhami.
Received 7 April 1989; and in revised form 18 September 1989.

**Table I** Numbers of patients admitted to studies by each participating centre, and overall proportion of patients surviving 2 years or more

| Trial centre | No. of trials | Patients entered on study: Total number | Patients entered on study: 2 year survivors (%) |
|---|---|---|---|
| 1. MRC | 5 | 1,139 | 66 (5.8) |
| 2. UCH/Brompton/LCH | 6 | 1,058 | 46 (4.3) |
| 3. Manchester | 4 | 434 | 37 (8.5) |
| 4. Midlands | 1 | 309 | 19 (6.1) |
| 5. Marsden | 4 | 277 | 16 (5.7) |
| 6. Edinburgh | 3 | 228 | 16 (7.0) |
| 7. Clatterbridge | 3 | 122 | 13 (10.6) |
| 8. Dublin | 4 | 114 | 4 (3.5) |
| Totals | 30 | 3,681 | 217 (5.9) |

**Table II** The design of studies contributing patients to the analysis

| Centre | Trial no. | Design | Drugs | Thoracic RT | No. of patients | No. of 2 year survivors |
|---|---|---|---|---|---|---|
| 1 | 1 | CT + RT vs RT | CY + ME/CCNU | LD | 237 | 16 |
| | 2 | CT (A) vs CT (B) | CY vs CY + ME + CCNU | – | 68 | 1 |
| | 3 | R + CT vs CT + RT + CT | CY + ME + CCNU | LD | 186 | 12 |
| | 4 | CT vs SELECTIVE | ET + CY + ME + V | LD | 151 | 8 |
| | 5 | CT (A) vs CT (B) | ET + CY + ME + V | LD | 497 | 29 |
| 2 | 1 | CT + RT vs CT | AD + V/CY + ME | LD + ED | 371 | 7 |
| | 2 | CT (A) vs CT (B) | CY + V + ET | –[c] | 610 | 23 |
| | 3 | CT + RT[a] | CY | LD | 26 | 8 |
| | 4 | CT + RT[a] | CY/CY + ET | LD[b] | 26 | 4 |
| | 5 | CT[a] | AD + ET + V/CY | – | 18 | 2 |
| | 6 | CT + RT[a] | C + ET/MEL or CY | LD[b] | 7 | 2 |
| 3 | 1 | CT + RT[a] | ME + CY | LD + ED | 65 | 6 |
| | 2 | CT + RT[a] | CY + ET + ME | LD + ED | 122 | 8 |
| | 3 | CT + RT[a] | CY + ET + ME | LD + ED | 79 | 5 |
| | 4 | CT + RT[a] | IF + ET | LD + ED | 168 | 18 |
| 4 | 1 | CT (A) vs CT (B) | V + AD + CY | LD[b] | 309 | 19 |
| 5 | 1 | CT[a] | CY + CC + ME | LD + ED | 53 | 4 |
| | 2 | CT (A) vs CT (B) | CY + ME + V | – | 45 | 1 |
| | 3 | CT[a] | CY/AD + ET + V | LD + ED[c] | 122 | 7 |
| | 4 | CT[a] | C + ET | LD | 57 | 4 |
| 6 | 1 | CT (A) vs CT (B) | ME + CY + CC + /– V + ME + CY + PR | LD | 83 | 8 |
| | 2 | CT + RT[a] | ME + CY + ET + /– MEL | LD + ED[b] | 97 | 6 |
| | 3 | | VIN + ET + /– TCNU | | 48 | 2 |
| 7 | 1 | CT + RT[a] | IF + ET | LD + ED[c] | 19 | 2 |
| | 2 | CT[a] | CY + AD + V/IF + ME + ET | LD[b] | 72 | 4 |
| | 3 | SURGERY + CT | CY + AD + /– ET | – | 31 | 7 |
| 8 | 1 | CT[a] | CY + AD + V | – | 67 | 3 |
| | 2 | CT[a] | CY + AD + ET | – | 23 | 1 |
| | 3 | CT[a] | CY | – | 12 | 0 |
| | 4 | SC | – | – | 12 | 0 |

[a]Non-randomised trials; [b]PCI in LD responders; [c]PCI in LD and ED responders. AD, adriamycin; V, vincristine; CC, CCNU; C, carboplatin; CY, cyclophosphamide; P prednisolone; CT, chemotherapy; ET, etoposide; MEL, melphalan; RT, thoracic radiotherapy; IF, ifosfamide; VIN, vindesine; SC, supportive care; ME, methotrexate.

**Table III** Numbers and characteristics of patients entered into studies, with proportions surviving 2 years

| Trial centre | No. of patients | Disease extent (L:E): All patients No. (%) | Disease extent (L:E): 2 year survivors No. (% of total) | Mean age: All | Mean age: 2 years | Male: female: All patients No. (%) | Male: female: 2 year survivors No. (% of total) |
|---|---|---|---|---|---|---|---|
| 1 | 1139 | 900 (79): 239 (21) | 64 (7.1) : 2 (1.0) | 60 | 56 | 784 (70): 355 (30) | 41 (5.2) : 25 (7.0) |
| 2 | 1058 | 402 (38): 656 (62) | 36 (9.0) : 10 (1.5) | 62 | 58 | 694 (66): 364 (34) | 29 (4.2) : 17 (4.7) |
| 3 | 434 | 257 (59): 177 (41) | 28 (10.9): 9 (5.1) | 58 | 58 | 301 (70): 133 (30) | 22 (7.3) : 15 (11.3) |
| 4 | 309 | 79 (23): 230 (77) | 11 (14.0): 8 (3.5) | 62 | 60 | 204 (66): 105 (34) | 13 (6.4) : 6 (5.7) |
| 5 | 277 | 131 (47): 146 (53) | 13 (10.0): 0 (0) | 62 | 61 | 187 (68): 90 (32) | 9 (4.8) : 7 (7.8) |
| 6 | 228 | 118 (52): 110 (48) | 11 (9.3) : 5 (4.5) | 63 | 61 | 142 (62): 86 (38) | 9 (6.3) : 7 (8.1) |
| 7 | 122 | 89 (73): 33 (27) | 13 (14.6): 0 (0) | 57 | 61 | 77 (63): 45 (37) | 9 (11.7): 4 (8.9) |
| 8 | 114 | 58 (51): 56 (49) | 4 (6.9) : 0 (0) | 63 | 55 | Unknown | 3 (–): 1 (–) |
| Total | 3681 | L 2034 (55) | 180 (8.5) | | | M 2388 (67) | 135 (5.7) |
| | | E 1,647 (45) | 37 (2.2) | | | F 1178 (33) | 82 (7.0) |

men and women surviving 2 years was not significantly different from the proportion entering the trials. The proportion of patients with limited or extensive disease treated was largely determined by the study design. For example, the majority of the MRC studies during this period were in patients categorised as having limited stage disease but the London group (group 2) included limited and extensive stage patients in their studies. Table IV gives the distribution of patients treated with chemotherapy and radiotherapy according to trial centre and disease extent. It can be seen that there is wide distribution of types of treatment between the centres so that the analysis of effects of treatment on survival are not dependent on the results of one or two very large trials.

The causes of death are shown in Table V. The majority of patients died from recurrent SCLC but 29 patients died from other diseases of which vascular disease and other respiratory disorders were the commonest. The cause of death is unknown in 15. Only three patients were recorded as dying of a second cancer, and leukaemia was not reported as a cause of death. However, a second cancer was reported in eight patients surviving more than 2 years, of which five were smoking-related (Table VI).

The survival analysis is presented from 2 years onwards and is expressed in two ways. The first includes all deaths (SCLC, other causes of death and unknowns), and the second deaths from SCLC alone where the few patients who died of unknown cause are removed and the curves are censored for deaths other than SCLC. Both analyses give very similar results. Overall survival is shown in Figure 1a. Figure 1b shows death from SCLC alone and Figure 2 non-SCLC causes of death. At 6 years 45% of the 2 year survivors are alive (3% of all patients). Death from SCLC (Figure 2) ceases at 7 years and patients can be considered cured beyond this point. The hazard of death from other causes (Figure 2) continues throughout the period of observation. Although a greater proportion of patients presenting with limited disease survived to 2 years, beyond 2 years survival was not statistically different in patients initially staged as limited or extensive (Figure 3a and b). Patients below the age of 55 at diagnosis had a slightly greater chance of survival beyond 2 years (Figure 4a) but this was not significant statistically and was in part due to a lesser risk of death from other causes and the risk of dying from SCLC alone is only slightly influenced by age (Figure 4b). Female sex was not associated with improved survival beyond 2 years (Figure 5a and b). Patients who were treated with thoracic radiotherapy did not have an increased chance of survival beyond 2 years (Figure 6a and b). There was a tendency for patients treated with prophylactic cranial irradiation to have a slightly better prognosis after 2 years (Figure 7a) but this was not apparent when SCLC deaths alone were considered (Figure 7b). Treatments were not of course assigned randomly in the whole population and any relation to prognosis may of course be due to bias in selecting cases for treatment.

**Table IV** Treatment modality used in the trials according to disease extent, with proportion surviving 2 years

| Trial centre | Total no. of patients | Chemotherapy alone No. | Chemotherapy alone 2 year (%) | Chemotherapy + thoracic radiation No. | Chemotherapy + thoracic radiation 2 year (%) |
|---|---|---|---|---|---|
| 1. | L 900 | 54 | 0 | 846 | 64(8) |
| | E 239 | 239 | 2(1) | 0 | 0 |
| 2. | L 402 | 302 | 17(6) | 100 | 19(19) |
| | E 656 | 540 | 7(1) | 116 | 3(3) |
| 3. | L 257 | 0 | 0 | 257 | 28(11) |
| | E 177 | 99 | 3(3) | 78 | 6(8) |
| 4. | L 79 | 0 | 0 | 79 | 11(14) |
| | E 230 | 230 | 8(3) | 0 | 0 |
| 5. | L 131 | 99 | 7(7) | 32 | 6(19) |
| | E 146 | 128 | 1(<1) | 18 | 2(1) |
| 6. | L 118 | 101 | 7(7) | 17 | 4(23) |
| | E 110 | 106 | 4(4) | 4 | 1(25) |
| 7. | L 89 | 69 | 8(12) | 20 | 5(25) |
| | E 33 | 27 | 0 | 6 | 0 |
| 8. | L 58 | 58 | 4(7) | 0 | 0 |
| | E 56 | 56 | 0 | 0 | 0 |
| Totals | L 2034 | 683 | 43(6) | 1351 | 137(10) |
| | E 1647 | 1425 | 25(2) | 222 | 12(5) |

**Table V** Causes of death in the 217 two year survivors

| *Causes of death* | *Number of patients* |
|---|---|
| 1. Small cell lung cancer | 77 |
| 2. Vascular disease | |
| Myocardial | 6 |
| Cerebral | 5 |
| 3. Second cancer | 3 |
| 4. Respiratory failure | 12 |
| 5. Other | 3 |
| 6. Unknown | 15 |
| 7. Alive | 96 |
| Total number | 217 |

**Table VI** New malignancies in the 217 two year survivors

| *Diagnosis* | *No. of months from start of chemotherapy* | *Sex* | *Dead/alive* |
|---|---|---|---|
| Basal cell carcinoma | 60 | M | A |
| Carcinoma of the bladder | 60 | M | D |
| Carcinoma of the bladder[a] | 48 | F | D |
| Squamous cell carcinoma lung[a] | 43 | M | D |
| Carcinoma of larynx | 36 | M | A |
| Carcinoma of breast | 34 | F | D |
| Carcinoma of colon[a] | 32 | F | D |
| Carcinoma of pancreas | 31 | M | D |

[a]Primary cause of death.

**Figure 1** Survival from 2 years. **a**, All causes of death included ($n = 217$). **b**, Deaths from SCLC alone (unknowns removed, death from non SCLC causes censored: $n = 199$).

**Figure 2** Deaths from SCLC (B) compared with non SCLC causes (A). Patients with unknown cause of death are removed from both analyses.

**Figure 3** Survival from 2 years related to initial disease extent. **a**, All causes of death included. Limited disease, L ($n = 180$). Extensive disease, E ($n = 37$). $P = 0.423$. **b**, Deaths from SCLC alone (censored for non-SCLC causes and patients with unknown cause of death removed from the analysis). Limited disease, L ($n = 163$). Extensive disease, E ($n = 36$). $P = 0.772$.

### Discussion

We believe this analysis gives a broadly accurate assessment of the results of contemporary treatment of SCLC in major trials centres in the UK. The proportions of all patients surviving at 2 and 6 years (5.9% and 3%) are identical with those reported by Osterlind *et al.* (1986) and Davis *et al.* (1985). Relapse and death from SCLC occurs beyond 2 years and reported 2 year survival data should not be regarded as indicating cure rate. Patients in treatment trials in SCLC (for

**Figure 4** Survival from 2 years related to age at diagnosis. **a**, Including all causes of death. A, $\leqslant 55$ years ($n = 72$); B, 56–65 years ($n = 96$); C $\geqslant 66$ years ($n = 47$). (A *vs* C, $P = 0.031$; $P$ value for trend = 0.024). **b**, Deaths from SCLC alone (censored for non SCLC causes and patients with unknown cause of death removed from the analysis). A, $\leqslant 55$ years ($n = 67$); B, 56–65 years ($n = 86$); C, $\geqslant 66$ years ($n = 44$). No significant difference in any comparison.

example those assessing the value of thoracic or cranial irradiation) should be followed for a minimum of 5 years to assess impact on long-term survival. Beyond this point death from SCLC is uncommon.

The survival data presented here, although demonstrating the bad long-term prognosis of the disease, do not indicate that treatment is not worthwhile. Some patients, mostly those with limited disease, are cured, albeit only a small number. This implies that a large number of patients were nearly cured by current treatment and justifies, and should encourage, trials of treatment strategies in that category of patients in whom cure is a realistic aim. These are mostly patients with limited disease. Other studies (Souhami *et al.*, 1985) have shown the additional importance of factors such as performance status and biochemistry in identification of patients with an increased chance of survival at 2 years.

It must be stressed that the data concern survival beyond two years. Other studies have shown the benefit of chemotherapy on median and one year survival (Ihde, 1984). The effect of thoracic irradiation on survival before 2 years is more controversial and may be influenced by dose and timing of treatment. It seems probable that there is a modest benefit in patients with limited disease (Perry *et al.*, 1987). The present data suggest that even if patients receiving thoracic radiation are more likely to reach 2 years, the impact of thoracic radiotherapy on survival beyond 2 years is minimal. Treatment trials in limited disease, which do not include thoracic irradiation, still seem justified so far as long-term survival is concerned. The same is true for the use of prophylactic cranial irradiation (PCI). Only long-term results of

**Figure 5** Survival related to sex. **a**, All causes of death included. Female, F ($n = 83$). Male, M ($n = 134$). $P = 0.671$. **b**, Deaths from SCLC alone (censored for non SCLC causes and patients with unknown cause of death removed from the analysis). Female, F ($n = 75$). Male, M ($n = 124$). $P = 0.940$.

**Figure 7** Survival related to prophylactic cranial irradiation (PCI). **a**, All causes of death. PCI + ($n = 52$). PCI − (no PCI, $n = 165$). $P = 0.163$. **b**, Deaths from SCLC alone (censored for non SCLC causes and patients with unknown cause of death removed from the analysis). PCI + ($n = 52$). PCI − ($n = 147$). $P = 0.313$.

very large randomised studies can ultimately show the effect of these measures, if any, but it is clearly likely to be small. The findings indicate that for the vast majority of patients treatment is palliative, with only 2.2% of patients with extensive disease (who constitute 70% of unselected cases) surviving 2 years. For most of these patients chemotherapy undoubtedly provides effective palliation of symptoms and will prolong short-term survival. Long-term toxicity of measures such as thoracic radiation and PCI was not recorded in the trial follow-up data.

Beyond 6 years other smoking-related diseases become the major cause of death, particularly chronic bronchitis, vascular disease and smoking-related cancer. We have not found any case of leukaemia. The accuracy of death certification may be at fault here, but it is noteworthy that very few studies used CCNU or procarbazine while these drugs have been used in most of the cases where acute leukaemia has occurred following SCLC treatment (Pedersen-Bjergaard *et al.*, 1986).

This survey indicates that cure is possible in SCLC but that we have a long way to go to improve results. Given the present results with conventional treatment, trials of novel and intensive treatments to increase cure rate are fully justified. Similarly, assessment of palliative regimens is an important area of futher study. Treatment trials should pres-

**Figure 6** Survival related to thoracic radiotherapy. **a**, All causes of death included. RT + , thoracic radiotherapy ($n = 149$). RT −, no thoracic radiotherapy ($n = 68$). $P = 0.236$. **b**, Deaths from SCLC alone (censored for non SCLC causes and patients with unknown cause of death removed from the analysis). RT + ($n = 135$). RT − ($n = 64$). $P = 0.355$.

ent 5 year survival data before claiming a benefit in long-term survival or cure rate. Beyond this point death from SCLC is very uncommon and the 5 year survival figure is a fairly accurate estimate of cure rate.

We are most grateful to the following for providing us with the information on which this report is based. Medical Research Council: Dr David Girling, Mr Richard Stephens; Wythenshawe Hospital: Dr Nicholas Thatcher, Ms Linda Ashcroft; Midlands: Dr Michael Cullen, Ms Charlotte Woodroffe, Dr David Morgan; Royal Marsden Hospital: Dr Ian Smith, Ms Julia Woodiwiss; Western General Hospital. Edingburgh: Professor John Smyth, Ms Moira Stewart; Clatterbridge Hospital: Dr John Green, Dr Simon Watkin; St James' Hospital and Permount Hospital, Dublin: Dr P.A Daly.

### References

BERGSAGEL, D. & FELD, R. (1984). Small cell lung cancer is still a problem. *J. Clin. Oncol.*, **2**, 1189.

DAVIS, S., WRIGHT, P.W., SCHULMAN, S.F., SCHOLES, D., THORNING, D. & HAMMAR, S. (1985). Long-term survival in small cell carcinoma of the lung: a population experience. *J. Clin. Oncol.*, **3**, 80.

FELD, R., EVANS, W.K., COY, P. & 6 others (1987). Canadian multicenter randomised trial comparing sequential and alternating administration of two non-cross-resistant chemotherapy combination in patients with limited small cell carcinoma of the lung. *J. Clin. Oncol.*, **5**, 1401.

GRECO, F.A., RICHARDSON, R.L. & OLDHAM, R.K. (179). Small cell lung cancer: complete remission and improved survival. *Am. J. Med.*, **66**, 625.

IHDE, D.C. (1984). Current status of therapy for small cell carcinoma of the lung. *Cancer*, **54**, 2722.

OSTERLIND, K., HANSEN, H.H., HANSEN, M. & DOMBERNOWSKY, P. (1986). Mortality and morbidity in long-term surviving patients treated with chemotherapy with or withour irradiation for small cell lung cancer. *J. Clin. Oncol.*, **4**, 1044.

PEDERSEN-BJERGAARD, J., OSTERLIND, K., HANSEN, M. *et al.* (1986). Secondary malignancies following intensive chemotherapy of small cell carcinoma of the lung. *Blood*, **66**, 1393.

PERRY, M.C., EATON, W.L., PROPERT, K.J. & 9 others (1987). Chemotherapy with or without radiation therapy in limited stage small cell carcinoma of the lung. *N. Engl. J. Med.*, **316**, 912.

PETO, R., PIKE, M.C., ARMITAGE, P. *et al.* (1977). Design and analysis of randomized clinical trials requiring prolonged observation of each patient. II. Analysis and examples. *Br. J. Cancer*, **35**, 1.

SOUHAMI, R.L., BRADBURY, I., GEDDES, D.M., SPIRO, S.G., HARPER, P.G. & TOBIAS, J.S. (1985). Prognostic significance of laboratory parameters measured at diagnosis in small cell carcinoma of the lung. *Cancer Res.*, **45**, 2878.